# DNA barcoding of black flies (Diptera: Simuliidae) in Indonesia

**DOI:** 10.1186/s13071-023-05875-1

**Published:** 2023-07-22

**Authors:** Yan Xin Hew, Zubaidah Ya’cob, Peter H. Adler, Chee Dhang Chen, Koon Weng Lau, Mohd Sofian-Azirun, Abdullah Halim Muhammad-Rasul, Qi Yan Putt, Noor Izwan-Anas, Upik Kesumawati Hadi, I. Wayan Suana, Hiroyuki Takaoka, Van Lun Low

**Affiliations:** 1 Tropical Infectious Diseases Research and Education Centre (TIDREC), Universiti Malaya, Kuala Lumpur, Malaysia; 2 Institute for Advanced Studies, Universiti Malaya, Kuala Lumpur, Malaysia; 3 Department of Plant and Environmental Sciences, Clemson University, Clemson, SC USA; 4 Institute of Biological Sciences, Faculty of Science, Universiti Malaya, Kuala Lumpur, Malaysia; 5 Entomology Laboratory, Division of Parasitology and Medical Entomology, School of Veterinary Medicine and Biomedical Sciences, IPB University, Bogor, Indonesia; 6 Faculty of Mathematics and Natural Science, University of Mataram (UNRAM), Mataram, Indonesia

**Keywords:** *Simulium*, Indonesia, COI, Phylogenetic

## Abstract

**Background:**

DNA barcoding is a valuable taxonomic tool for rapid and accurate species identification and cryptic species discovery in black flies. Indonesia has 143 nominal species of black flies, but information on their biological aspects, including vectorial capacity and biting habits, remains underreported, in part because of identification problems. The current study represents the first comprehensive DNA barcoding of Indonesian black flies using mitochondrial cytochrome *c* oxidase subunit I (COI) gene sequences.

**Methods:**

Genomic DNA of Indonesian black fly samples were extracted and sequenced, producing 86 COI sequences in total. Two hundred four COI sequences, including 118 GenBank sequences, were analysed. Maximum likelihood (ML) and Bayesian inference (BI) trees were constructed and species delimitation analyses, including ASAP, GMYC and single PTP, were performed to determine whether the species of Indonesian black flies could be delineated. Intra- and interspecific genetic distances were also calculated and the efficacy of COI sequences for species identification was tested.

**Results:**

The DNA barcodes successfully distinguished most morphologically distinct species (> 80% of sampled taxa). Nonetheless, high maximum intraspecific distances (3.32–13.94%) in 11 species suggested cryptic diversity. Notably, populations of the common taxa *Simulium* (*Gomphostilbia*) *cheongi*, *S.* (*Gomphostilbia*) *sheilae*, *S.* (*Nevermannia*) *feuerborni* and *S.* (*Simulium*) *tani* in the islands of Indonesia were genetically distinct from those on the Southeast Asian mainland (Malaysia and Thailand). Integrated morphological, cytogenetic and nuclear DNA studies are warranted to clarify the taxonomic status of these more complex taxa.

**Conclusions:**

The findings showed that COI barcoding is a promising taxonomic tool for Indonesian black flies. The DNA barcodes will aid in correct identification and genetic study of Indonesian black flies, which will be helpful in the control and management of potential vector species.

**Graphical abstract:**

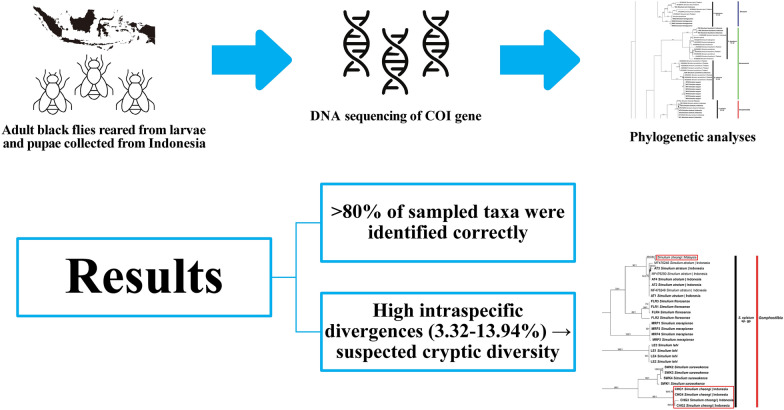

**Supplementary Information:**

The online version contains supplementary material available at 10.1186/s13071-023-05875-1.

## Background

Black flies (Diptera: Simuliidae) are medically important haematophagous insects for humans, domestic animals and wildlife, due to their pestiferous biting habits and vectorial roles in transmitting various parasites. They are the sole vector of the filarial nematode *Onchocerca volvulus*, which causes river blindness, the second leading infectious cause of blindness in the world [1]. They also transmit other *Onchocerca* species, *Mansonella* filarial parasites and *Leucocytozoon* and *Trypanosoma* protozoa [2, 3]. In contrast, black flies also function as beneficial organisms in aquatic ecosystems, where the larvae process fine particulate organic matter into larger food pellets, serve as food for other aquatic organisms and act as bioindicators of water quality [4].

Southeast Asia harbours nearly 20% of the world’s species of black flies, providing excellent opportunities for research on these minute creatures. The extensive morphotaxonomic research on black flies in Indonesia began in the late 1990s, leading to a total of 143 species reported from the country to date [5, 6]. The rich black fly biodiversity in Indonesia reflects its strategic location in the tropical belt between the Pacific and Indian Oceans and between the Asian and Australian continents. All Indonesian black flies are in the genus *Similium* Latreille and are classified in five subgenera: *Gomphostilbia* Enderlein, *Morops* Enderlein, *Nevermannia* Enderlein, *Simulium* Latreille and *Wallacelum* Takaoka. The species are further assigned to 27 species groups [6, 7]. Nevertheless, various biological aspects of black flies in Indonesia, including their vectorial roles and biting habits, remain to be explored. Exceptions include *S.* (*G.*) *atratum*, which bites domestic fowls in Java [8], and *S.* (*N.*) *aureohirtum*, which is autogenous [9, 10].

Black flies are traditionally identified using morphological keys, such as those by Adler, Currie [11], Crosskey [12], Shelley [13], Takaoka [14], Takaoka and Davies [15], Takaoka and Davies [16] and Takaoka, Sofian-Azirun [17]. Chromosome-based analyses also drive black fly taxonomy and have revealed cryptic diversity in many morphospecies [2]. These two methods, however, are sometimes insufficient for rapid and accurate species identification crucial for biological research and vector control. Morphologically similar species often cannot be differentiated in one or more life stages, and chromosomal identifications are typically applicable only in the larval stage. Both methods also require a higher level of expertise [18, 19].

The DNA barcoding approach has shown promise as a molecular taxonomic tool for black flies. Many DNA barcoding studies, based on the mitochondrial cytochrome *c* oxidase subunit I (COI) gene, demonstrate high levels of correct species identifications, which are usually consistent with morphotaxonomic and chromosomal studies. COI-based barcoding has demonstrated a considerable success level (> 90% sampled taxa) in distinguishing species of black flies from Thailand [20, 21]. The molecular approach is also helpful in revealing cryptic diversity in morphospecies thought to be single species. Thailand, in particular, has been actively reporting species complexes such as *S.* (*G.*) *angulistylum* Takaoka & Davies [22], *S.* (*N.*) *feuerborni* [23, 24] and *S.* (*S.*) *fenestratum* [25] through integrated initiatives of barcoding and cytogenetics. Coupled with other taxonomic approaches, DNA barcoding also complements the description of cryptic species. Some notable examples include the description of *S.* (*N.*) *pairoti* from *S.* (*N.*) *feuerborni* [26] and the naming of *S.* (*S.*) *nobile* cryptic species in Peninsular Malaysia as *S.* (*S.*) *vanluni* [27]. Additionally, *S.* (*S.*) *rufibasis* Brunetti in Japan and Korea was revised as *S.* (*S.*) *yamatoense* Takaoka, Adler & Fukuda after the morphological, chromosomal and molecular re-examinations of the species [28]. In the meantime, ongoing molecular research on these simuliids  is being carried out in Malaysia and Vietnam, hoping to contribute to the growing body of knowledge in this area.

Although several genetic studies have been conducted on black flies in Indonesia, including *S.* (*N.*) *feuerborni, S.* (*S.*) *nobile* and *S.* (*S.*) *timorense* [24, 29, 30], the genetics of other Indonesian black flies is understudied. We, therefore, used the mitochondrial COI gene to delimit species boundaries for 55 species of black flies from Indonesia.

## Methods

### Sample collection

Samples were collected from eight provinces in Indonesia between 2014 and 2017 (Table 1). Aquatic stages of black flies (larvae and pupae) attached to grasses, leaves, twigs, plant roots and rocks were collected by hand using fine forceps. Pupae were individually kept alive in vials until adult emergence. The adults, together with their pupal exuviae and cocoons, were fixed in 80% ethanol for identification at the subgenus, species group or species level. The methods of collection and identification followed those of Adler, Currie [11] and Takaoka [14].

**Table 1:** Black flies (*n* = 27) of Indonesia included in the present study of COI barcoding, with collection data and GenBank accession numbers

Species group/species	*n*	Sampling location	Latitude/longitude	Sampling date	GenBank accession no.
Subgenus *Gomphostilbia* Enderlein					
* Simulium asakoae* group					
* S. gyorkosae* Takaoka & Davies	2	Kakek Bodo, Tretes	N/A	18 Apr 2015	OQ117897-900
	1	Munduk, Bali	08°15′26.684″S/115°04′12.625″E	28 Sep 2014	
	1	Otak Kokok Joben, Lombok	08°31′57.775″S/116°23′51.041″E	25 Sep 2014	
* S. sunapii* Takaoka, Sofian-Azirun & Wayan	1	Rangat, Kempo, Flores^2^	08°36′20.443″S/120°01′19.156″E	28 Feb 2016	OQ117901
*Simulium batoense* group					
* S. lemborense* Takaoka & Sofian-Azirun	2	Mbatakapidu, Waingapu, Sumba	09°42′02.589″S/120°13′21.775″E	7 Oct 2017	OQ117902-05
	2	Mbatakapidu, Waingapu, Sumba	09°40′57.446″S/120°13′50.675″E	7 Oct 2017	
* S. tahanense* Takaoka & Davies	2	Long Ikis, East Kalimantan	00°33′04.414″S/116°06′01.340″E	3 Sep 2015	OQ117906-09
	2	Long Ikis, East Kalimantan	01°33′28.150″S/116°05′44.395″E	3 Sep 2015	
*Simulium ceylonicum* group					
* S. rangatense* Takaoka, Sofian-Azirun & Wayan^1^	1	Rangat, Kempo, Flores^2^	08°36′31.889″S/120°01′06.248″E	28 Feb 2016	OQ117910
* S. sheilae* Takaoka & Davies	4	Lembah Harau, West Sumatra	00°06′35.532″S/100°40′17.212″E	23 Nov 2016	OQ117911-14
*Simulium epistum* group					
* S. atratum* De Meijere	4	Suranadi, Narmada, Lombok	08°34′15.315″S/116°13′54.831″E	22 Sep 2014	OQ117915-18
* S. cheongi* Takaoka & Davies	4	East Kalimantan	N/A	3 Sep 2015	OQ117919-22
* S. floresense* Takaoka, Hadi & Sigit	1	Wae Garit, Ruteng, Flores^2^	08°35′27.877″S/120°26′05.799″E	27 Feb 2016	OQ117923-26
	1	Ruang, Ruteng, Flores	N/A	27 Feb 2016	
	1	Rangat, Kempo, Flores^2^	N/A	28 Feb 2016	
	1	Roe, Cunca Lolos, Flores	N/A	28 Feb 2016	
* S. lehi* Takaoka	3	East Kalimantan	N/A	4 Sep 2015	OQ117927-30
	1	Long Ikis, East Kalimantan	01°32′56.079″S/116°03′19.814″E	3 Sep 2015	
* S. merapiense* Takaoka & Sofian-Azirun	4	Taman Nasional Gunung Merapi, Kaliurang, Yogyakarta^2^	07°35′35.1″S/110°25′58.0″E	28 May 2014	OQ117931-34
* S. sarawakense* Takaoka	4	East Kalimantan	N/A	3 Sep 2015	OQ117935-38
*Simulium varicorne* group					
* S. sumbaense* Takaoka & Suana	1	Watumbaka, Waingapu, Sumba^2^	09°39′57.329″S/120°20′57.790″E	7 Oct 2017	OQ117939
Subgenus *Nevermannia* Enderlein					
* Simulium feuerborni,* group					
* S. feuerborni* Edwards	3	Kakek Bodo, Tretes	N/A	18 Apr 2015	OQ117940-42
*Simulium ruficorne* group					
* S. aureohirtum* Brunetti	1	Lembah Harau, West Sumatra	00°06′35.532″S/100°40′17.212″E	18 Apr 2015	OQ117943
* S. wayani* Takaoka & Chen	7	Boentuka, Timor	09°55′39.6″S/124°10′38.7″E	10 Oct 2017	OQ117944-51
	1	Polen, Soë, Timor^2^	09°41′32.117″S/124°28′57.748″E	11 Oct 2017	
Subgenus *Simulium* Latreille					
* Simulium eximium* group					
* S. eximium* De Meijere	1	Malang	N/A	17 Apr 2015	OQ117952-54
	1	Kakek Bodo, Tretes	N/A	18 Apr 2015	
	1	Wae Garit, Ruteng, Flores	08°35′27.877″S/120°26′05.799″E	27 Feb 2016	
*Simulium iridescens* group					
* S. iridescens* De Meijere	1	Munduk, Bali	08°15′26.684″S/115°04′12.625″E	28 Sep 2014	OQ117955-58
	1	Nungnung Waterfall, Bali	08°19′42.039″S/115°13′44.116″E	29 Sep 2014	
	1	Malang	N/A	17 Apr 2015	
	1	Kakek Bodo, Tretes	N/A	18 Apr 2015	
* S. javaense* Takaoka & Hadi	2	Sebatu, Bali	08°23′56.592″S/115°17′52.803″E	27 Sep 2014	OQ117959-62
	2	Tampaksiring, Bali	08°24′55.853″S/115°18′55.844″E	27 Sep 2014	
*Simulium multistriatum* group					
* S. fenestratum* Edwards	1	West Sumatra	00°57′39.252″S/100°36′46.961″E	24 Nov 2016	OQ117963
*Simulium nebulicola* group					
* S. nebulicola* Edwards	1	Nte’er, Manggarai, Flores	08°41′03.955″S/120°19′17.135″E	27 Feb 2016	OQ117964
*Simulium nobile* group					
* S. nobile* De Meijere	2	West Sumatra	00°57′39.252″S/100°36′46.961″E	24 Nov 2016	OQ117965-68
	1	Sebatu, Bali	08°23′56.592″S/115°17′52.803″E	27 Sep 2014	
	1	Pusat Pendidikan Lingkungan Hidup (PPLH), Surubaya	N/A	16 Apr 2015	
* S. timorense* Takaoka, Hadi & Sigit	1	Wae Garit, Ruteng, Flores	N/A	27 Feb 2016	OQ117969-72
	1	Narmada, Lombok	08°35′46.850″S/116°12′18.327″E	23 Sep 2014	
	1	Sungai Toloweri, Nunger, Bima, Sumbawa	N/A	21 Feb 2016	
	1	Mbatakapidu, Waingapu, Sumba	09°42″02.589″S/120°13′21.775″E	7 Oct 2017	
*Simulium striatum* group					
* S. argyrocinctum* De Meijere	2	Coban Talun, Malang	N/A	17 Apr 2015	OQ117973-76
	1	Puncak, Bogor	N/A	14 Apr 2015	
	1	Pusat Pendidikan Lingkungan Hidup (PPLH), Surubaya	N/A	16 Apr 2015	
* S. baliense* Takaoka & Sofian-Azirun	1	Sebatu, Bali	08°23′56.592″S/115°17′52.803″E	27 Sep 2014	OQ117977
*Simulium tuberosum* group					
* S. keningauense* Takaoka	4	East Kalimantan	N/A	3 Sep 2015	OQ117978-81
* S. tani* Takaoka & Davies	1	West Sumatra	00°57′39.252″S/100°36′46.961″E	24 Nov 2016	OQ117982

^1^*Simulium rangatense* is represented by a type specimen

^2^Specimens were collected from type localities

### DNA extraction, polymerase chain reaction (PCR) and sequencing

One to four adults were selected randomly and dissected for each species before DNA extraction. Genomic DNA was extracted from the dissected parts (thorax or hind leg), using the NucleoSpin^®^ Tissue Mini Kit (Macherey–Nagel, Düren, Germany), according to the manufacturer’s protocol. A conventional polymerase chain reaction (PCR) was then performed to amplify the target region of the cytochrome *c* oxidase subunit I (COI) gene, using the DNA barcoding standard primers: LCO1490 (5′-GGTCAACAAATCATAAAGATATTGG-3′) and HCO2198 (5′-TAAACTTCAGGGTGACCAAAAAATCA-3′) [31]. Each PCR reaction mixture contained 1 µl DNA template, 12.5 µl MyTaq^™^ Red Mix 2 × mastermix (Bioline Reagents, Meridian Bioscience, Cincinnati, Ohio, USA), 0.4 µM forward primer, 0.4 µM reverse primer and distilled water up to 25 µl. The PCR amplifications were performed on Applied Biosystems Veriti 96-Well Thermal Cycler (Applied Biosystems, Inc., Foster City, CA, USA). PCR reaction conditions and temperature profiles followed those of Rivera and Currie [19]: denaturation at 96 °C for 1 min and 94 °C for 1 min, primer annealing at 55 °C for 1 min, 35 cycles of amplification at 72 °C for 1.5 min and 7 min at 72 °C. PCR products were visualized on a 1.5% agarose gel electrophoresis pre-stained with SYBR Safe dye (Invitrogen Corp., Carlsbad, CA, USA) run using a 100-bp DNA ladder (GeneDireX, Inc., Taiwan) as the DNA band size standard. Lastly, the PCR amplicons were sent to Apical Scientific Sdn Bhd (Selangor, Malaysia) for sequencing.

### Data analyses

Publicly available COI sequences of other related black fly species were retrieved from the NCBI GenBank database and included in analyses. A total of 204 black fly COI sequences representing 55 species from 14 species groups were analysed, with 86 of the sequences generated in the present study. Representative sequences were deposited in the NCBI GenBank database under accession numbers OQ117897–OQ117982 and the Global Biodiversity Information Facility (GBIF) database with other relevant information. The COI sequences were aligned in Unipro UGENE software using MUSCLE [32] and were trimmed to 452 bp in BioEdit software [33]. Before phylogenetic analyses, model selection was performed using kakusan4 to determine the most suitable nucleotide substitution model [34]. Trees were constructed based on the COI sequences via maximum-likelihood (ML) and Bayesian inference (BI) methods. *Parasimulium crosskeyi* (GenBank accession number: FJ524489) [21] was chosen as an outgroup for both tree analyses. The ML tree was generated from RAxML webserver (https://raxml-ng.vital-it.ch/#/) [35] using a generalized time-reversible (GTR) nucleotide substitution model with invariant sites of 0.47 (I), a gamma shape parameter (α) of 0.56 (G), four mean gamma category rates and maximum likelihood search. Bootstrap support was estimated for 100 replicates. The configuration file generated from kakusan4 was used to perform BI tree analysis using MrBayes v3.2.7 [36] on CIPRES Science Gateway v3.3 webserver (https://www.phylo.org/portal2/home.action). The BI analysis adopted the GTR substitution model using gamma-distributed rate variation across sites with shape parameter of 0.767 and invariable sites of 0.466. The posterior probability distribution of trees was estimated from two independent Markov chain Monte Carlo (MCMC) simulations of five million generations until the average standard deviation of split frequencies reached < 0.01. The first 25% of all runs was discarded as burn-in.

Species delimitation analyses, including Assemble Species by Automatic Partitioning (ASAP) [37], Generalized Mixed Yule Coalescent (GMYC) [38] and single Poisson Tree Processes (PTP) [39], were also performed. ASAP analysis was performed in the webserver version (https://bioinfo.mnhn.fr/abi/public/asap/). The Jukes-Cantor (JC69), Kimura (K80) ts/tv and simple distance models were tested. Results with genetic distances between 0 and 0.03 were highlighted. The GMYC analysis adopted an ultrametric tree generated from BEAUti2 software using a GTR + G + I model, Yule prior and relaxed clock log-normal model. The analysis was run for 40 million generations with a sampling frequency of every 1000 generations in BEAST v2.6.7. The output file was visualised using Tracer v1.6 software to ensure all estimated sample sizes (ESS) of all parameters exceeded 200. The output tree was then analysed in TreeAnnotator v2.6.7 software with a 20% burn-in. Data were analysed using a single threshold model in the SPLITS software package [40] available in the R v3.3.0 program. The single PTP analysis was performed in the mPTP webserver (https://mptp.h-its.org/#/tree) with the tree obtained from RAxML as input file and PTP with default p-value selected as the model for analysis with default settings. The intra- and interspecific genetic distances were calculated based on an uncorrected p-distance model with variance estimation using the bootstrap method for 1000 replicates in MEGA11 software [41]. Lastly, the efficacy of COI sequences for species identification was tested using the best match (BM) and best close match (BCM) methods in TaxonDNA software. The criterion for successful identifications based on the BM method was that all conspecifics had the smallest distance to the query sequence, whereas the BCM method required that the smallest distance be within the 95th percentile of overall intraspecific distances [42]. Using an *adhoc* R package [43], the cut-off threshold of BCM method was 1.9%.

## Results

### Phylogenetic analysis based on COI barcodes

Both ML and BI trees showed similar topologies. The only difference was in the placement of the *S.* (*S.*) *eximium* clade. *Simulium* (*Simulium*) *eximium* grouped with the *S.* (*S.*) *iridescens* group in the ML tree, whereas it clustered with the *S.* (*S.*) *multistriatum* group in the BI tree; only the ML tree is shown. The BI tree was included as a supplementary figure (see Additional file 1).

Three major clades were formed in the tree, corresponding to (i) subgenus *Simulium*, (ii) subgenera *Gomphostilbia* and *Nevermannia* and (iii) *Simulium* (*Gomphostilbia*) *tahanense*. Overall, most nominal species formed clades in their respective subgenera and species groups, consistent with morphotaxonomic studies, except for *S.* (*G.*) *tahanense*, which formed a distinct clade with strong bootstrap and posterior probability values.

### Subgenus *Simulium* Latreille

**Fig. 1:**
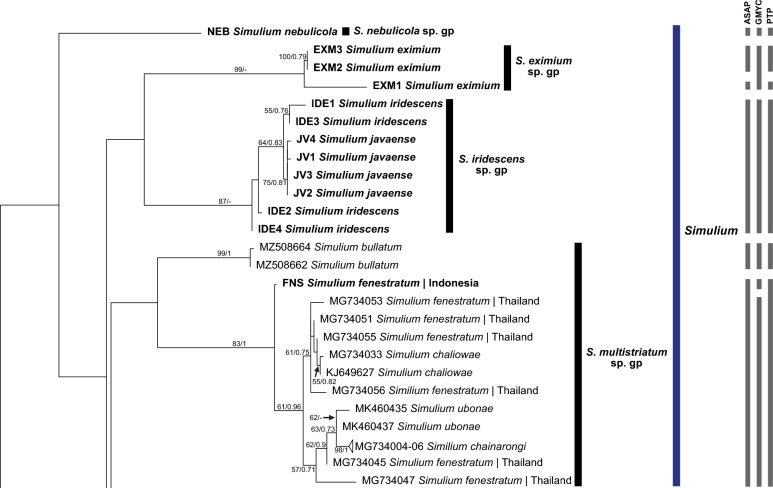
ML tree showing species of black flies from Indonesia in the subgenus *Simulium * Latreille, which was constructed from COI sequences. Bootstrap and posterior probability values of  > 50% and  > 0.50, respectively, are shown on the branches. Branches with bootstrap and posterior probability values > 70% and > 0.70, respectively, are considered well supported. New sequences generated in the study are in bold. Grey bars indicate the respective operational taxonomic units recognized by the three species delimitation analyses (i.e. ASAP, GMYC and PTP, in order). *ASAP* Assemble Species by Automatic Partitioning, *GMYC* Generalized Mixed Yule Coalescent, *PTP* Poisson Tree Processes

**Fig. 2:**
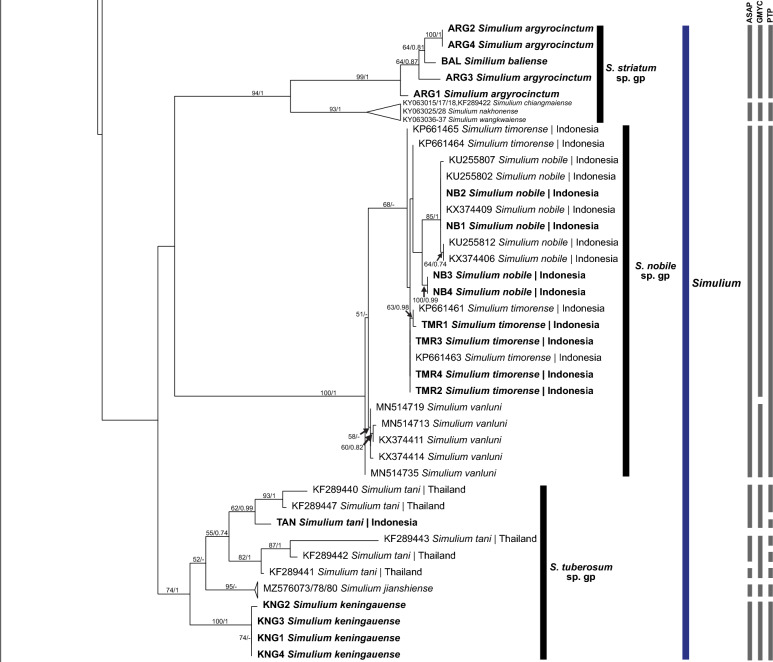
Continued ML tree showing species of black flies from Indonesia in the subgenus *Simulium* Latreille, which was constructed from COI sequences. Bootstrap and posterior probability values of  > 50% and  > 0.50, respectively, are shown on the branches. Branches with bootstrap and posterior probability values > 70% and > 0.70, respectively, are considered well supported. New sequences generated in the study are in bold. Grey bars indicate the respective operational taxonomic units recognized by the three species delimitation analyses (i.e. ASAP, GMYC and PTP, in order). *ASAP* Assemble Species by Automatic Partitioning, *GMYC* Generalized Mixed Yule Coalescent, *PTP* Poisson Tree Processes

All species groups of the subgenus *Simulium* were monophyletic (Figs. 1, 2). *Simulium nebulicola* was the only member of the *S. nebulicola* group represented in our study. It formed a distinct clade from other *Simulium* species groups with high interspecific distances. *Simulium eximium* formed a strongly supported clade, whereas *S. iridescens* was paraphyletic with the *S. javaense* clade nested in its clade. In the *S. multistriatum* group, *S. bullatum* formed a strongly supported distinct subclade. *Simulium fenestratum* formed two subgroups representing the only species in a distinct Indonesia group and a Thailand group that included the remaining members of the *S. multistriatum* group (*S. chainarongi, S. chaliowae* and *S. ubonae*). Within the *S. striatum* group, *S. argyrocinctum* was paraphyletic, with *S. baliense* nested within its clade. *Simulium chaingmaiense, S. nakhonense* and *S. wangkwaiense* formed a non-monophyletic clade with low genetic distances among these taxa. In the *S. nobile* group, one sequence of *S. vanluni* was distinct from the others that formed a separate clade of *S. vanluni*. The *S. nobile* clade was nested within the *S. timorense* clade, with low interspecific distances (minimum = 1.11%), making the *S. timorense* clade paraphyletic. However, in the BI tree, the *S. nobile* and *S. timorense* clades were well separated. In the *S. tuberosum* group, *S. jianshiense* and *S. keningauense* each formed a monophyletic clade, whereas *S. tani* was divided into two subgroups.

### Subgenus *Nevermannia* Enderlein

**Fig. 3:**
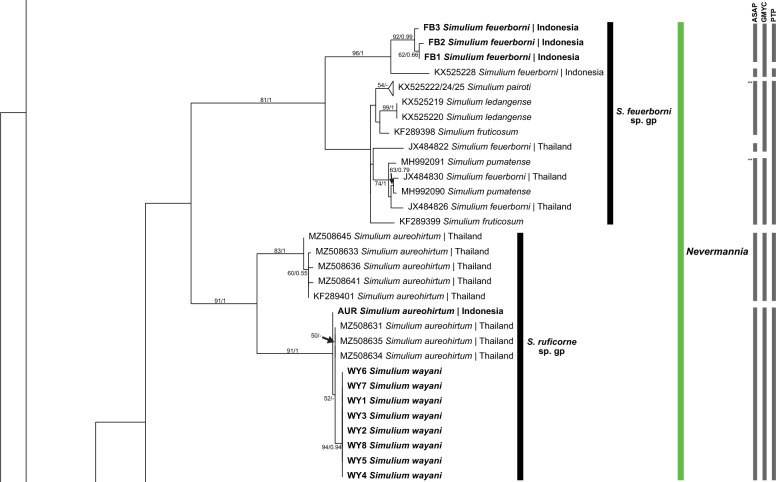
ML tree showing species of black flies from Indonesia in the subgenus *Nevermannia* Enderlein, which was constructed from COI sequences. Bootstrap and posterior probability values of  > 50% and  > 0.50, respectively, are shown on the branches. Branches with bootstrap and posterior probability values > 70% and > 0.70, respectively, are considered well supported. New sequences generated in the study are in bold. Grey bars indicate the respective operational taxonomic units recognized by the three species delimitation analyses (i.e. ASAP, GMYC and PTP, in order). The double asterisk (**) on the two grey bars of the ASAP analysis indicates these two bars represent the same taxonomic unit. *ASAP* Assemble Species by Automatic Partitioning, *GMYC* Generalized Mixed Yule Coalescent, *PTP* Poisson Tree Processes

In subgenus *Nevermannia*, two clades formed representing the *S. feuerborni* species group and the *S. ruficorne* species group (Fig. 3). Members of the *S. feuerborni* group were divided into two subgroups, showing the paraphyly of *S. feuerborni* with other taxa. The two subgroups corresponded to *S. feuerborni* from Indonesia and Thailand, which were non-monophyletic with other members of the *S. feuerborni* group (*S. fruticosum, S. ledangense, S. pairoti* and *S. pumatense*). In the *S. ruficorne* group, *S. aureohirtum* was divided into two subgroups of which one subgroup had sequences of *S. wayani* nested within.

### Subgenus *Gomphostilbia* Enderlein

**Fig. 4:**
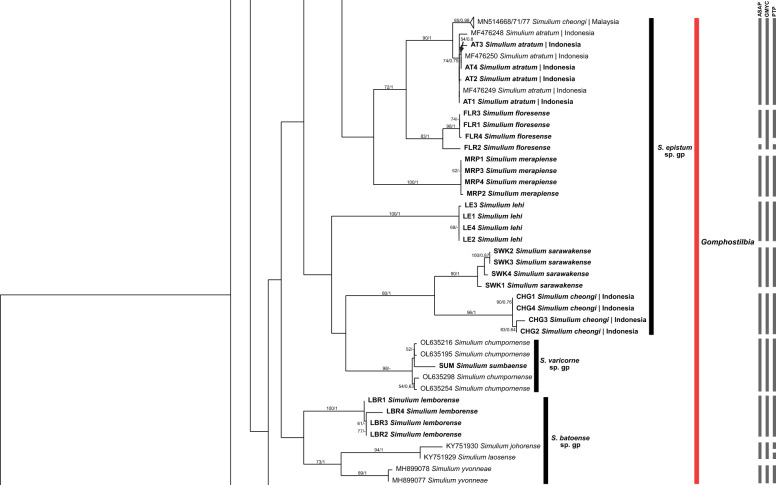
ML tree showing species of black flies from Indonesia in the subgenus *Gomphostilbia* Enderlein, which was constructed from COI sequences. Bootstrap and posterior probability values of  > 50% and > 0.50, respectively, are shown on the branches. Branches with bootstrap and posterior probability values > 70% and > 0.70, respectively, are considered well supported. New sequences generated in the study are in bold. Grey bars indicate the respective operational taxonomic units recognized by the three species delimitation analyses (i.e. ASAP, GMYC, and PTP, in order). *ASAP* Assemble Species by Automatic Partitioning, *GMYC* Generalized Mixed Yule Coalescent, *PTP* Poisson Tree Processes

**Fig. 5:**
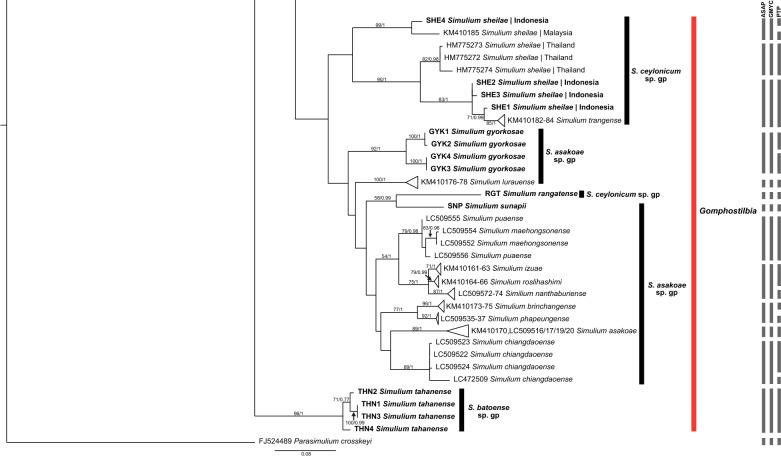
Continued ML tree showing species of black flies from Indonesia in the subgenus *Gomphostilbia* Enderlein, which was constructed from COI sequences. Bootstrap and posterior probability values of  > 50% and > 0.50, respectively, are shown on the branches. Branches with bootstrap and posterior probability values > 70% and > 0.70, respectively, are considered well supported. New sequences generated in the study are in bold. Grey bars indicate the respective operational taxonomic units recognized by the three species delimitation analyses (i.e. ASAP, GMYC and PTP, in order). *ASAP* Assemble Species by Automatic Partitioning, *GMYC* Generalized Mixed Yule Coalescent, *PTP* Poisson Tree Processes

The nominal species of the subgenus *Gomphostilbia* formed two clades: a major clade with subgenus *Nevermannia* clustering with the *Simulium epistum* group and a strongly supported distinct *S. tahanense* clade of the *S. batoense* group (Figs. 4, 5). Other members of the *S. batoense* group were monophyletic.

The *S. asakoae* group was not monophyletic. It had a member of the *S. ceylonicum* group (*S. rangatense*) clustering with one of its members (*S. sunapii*)*.* Nonetheless, the high genetic distance (8.85%) between *S. rangatense* and *S. sunapii* suggests that they are distinct species. Other taxa of the *S. asakoae* group formed a monophyletic clade except for *S. puaense*, which contained *S. maehongsonense* in the ML tree. In the BI tree, however, all members of the *S. asakoae* group were monophyletic.

In the *S. ceylonicum* group, *S. sheilae* was paraphyly because its clade included *S. trangense*. This clade was further divided into three subclades: (i) Malaysia and Indonesia, (ii) Thailand and (iii) Indonesia + *S. trangense*. The *S. epistum* species group formed four subclades: (i) *S. cheongi* (Malaysia), *S. atratum* and *S. floresense*; (ii) *S. merapiense*; (iii) *S. lehi*; (iv) *S. sarawakense* and *S. cheongi* (Indonesia). All involved taxa were monophyletic, except for *S. cheongi*. *Simulium chumpornense* and *S. sumbaense* of the *S. varicorne* group formed a paraphyletic clade, clustering with subclade iv of the *S. epistum* group.

### Genetic distances

The maximum intraspecific genetic distance ranged from 0% in *S.* (*N.*) *ledangense, S.* (*N.*) *wayani* and *S.* (*S.*) *chainarongi* to 13.94% in *S.* (*G.*) *cheongi.* Out of 55 morphospecies, 11 exhibited high intraspecific divergences, with mean and maximum values reported as follows: *S.* (*G.*) *gyorkosae* (2.18%; 3.32%), *S.* (*G.*) *sheilae* (5.75%; 9.51%), *S.* (*G.*) *cheongi* (7.93%; 13.94%), *S.* (*G.*) *floresense* (1.88%; 3.76%), *S.* (*N.*) *feuerborni* (7.04%; 10.62%), *S.* (*N.*) *aureohirtum* (4.36%; 7.96%), *S.* (*S.*) *eximium* (2.51%; 3.76%), *S.* (*S.*) *iridescens* (2.14%; 3.32%), *S.* (*S.*) *fenestratum* (2.58%; 4.42%), *S.* (*S.*) *argyrocinctum* (2.80%; 3.54%) and *S.* (*S.*) *tani* (5.32%; 7.74%) (Table 2). Among these species, *S.* (*N.*) *feuerborni, S.* (*S.*) *fenestratum and S.* (*S.*) *tani* are known to be species complexes.

**Table 2:** Species of black flies (*n* = 55) included for barcoding analyses (*n* = 204 COI sequences), with the mean and maximum intraspecific divergence values (%) of each species

Subgenus	Species group	Species	*n* (from this study)	Mean intraspecific divergence (maximum), %
*Gomphostilbia* Enderlein	*S. asakoae*	*S. asakoae* Takaoka & Davies	5	1.59 (2.43)
		*S. brinchangense* Takaoka, Sofian-Azirun & Hashim	3	0.44 (0.66)
		*S. chiangdaoense* Takaoka & Srisuka	4	1.22 (2.21)
		*S. gyorkosae* Takaoka & Davies	(4)	2.18 (3.32)
		*S. izuae* Takaoka, Sofian-Azirun & Hashim	3	0.74 (0.88)
		*S. lurauense* Takaoka, Sofian-Azirun & Hashim	3	0.88 (1.11)
		*S. maehongsonense* Takaoka, Srisuka & Saeung	2	–
		*S. nanthaburiense* Takaoka, Srisuka & Fukuda	3	0.59 (0.66)
		*S. phapeungense* Takaoka, Srisuka & Fukuda	3	0.29 (0.44)
		*S. puaense* Takaoka, Srisuka & Saeung	2	–
		*S. roslihashimi* Takaoka & Sofian-Azirun	3	0.29 (0.44)
		*S. sunapii* Takaoka, Sofian-Azirun & Wayan	(1)	–
	*S. batoense*	*S. johorense* Takaoka, Sofian-Azirun & Ya’cob	1	–
		*S. laosense* Takaoka, Srisuka & Saeung	1	–
		*S. lemborense* Takaoka & Sofian-Azirun	(4)	0.88 (1.77)
		*S. tahanense* Takaoka & Davies	(4)	1.33 (1.99)
		*S. yvonneae* Takaoka & Low	2	–
	*S. ceylonicum*	*S. rangatense* Takaoka, Sofian-Azirun & Wayan	(1)	–
		*S. sheilae* Takaoka & Davies	4 (4)	5.75 (9.51)^3^
		*S. trangense* Jitklang, Kuvangkadilok, Baimai, Takaoka & Adler	3	0.88 (1.33)
	*S. epistum*	*S. atratum* De Meijere	3 (4)	0.63 (1.77)
		*S. cheongi* Takaoka & Davies	3 (4)	7.93 (13.94)^3^
		*S. floresense* Takaoka, Hadi & Sigit	(4)	1.88 (3.76)
		*S. lehi* Takaoka	(4)	0.11 (0.22)
		*S. merapiense* Takaoka & Sofian-Azirun	(4)	0.11 (0.22)
		*S. sarawakense* Takaoka	(4)	1.33 (1.99)
	*S. varicorne*	*S. chumpornense* Takaoka & Kuvangkadilok	4	0.77 (1.11)
		*S. sumbaense* Takaoka & Suana	(1)	–
*Nevermannia* Enderlein	*S. feuerborni*	*S. feuerborni* Edwards	4 (3)	7.04 (10.62)^3^
		*S. fruticosum* Takaoka & Choochote	2	–
		*S. ledangense* Ya’cob, Takaoka & Sofian-Azirun	2	–
		*S. pairoti* Ya’cob, Takaoka & Sofian-Azirun	3	0.29 (0.44)
		*S. pumatense* Takaoka, Low & Pham	2	–
	*S. ruficorne*	*S. aureohirtum* Brunetti	8 (1)	4.36 (7.96)^3^
		*S. wayani* Takaoka & Chen	(8)	0
*Simulium* Latreille	*S. eximium*	*S. eximium* De Meijere	(3)	2.51 (3.76)
	*S. iridescens*	*S. iridescens* De Meijere	(4)	2.14 (3.32)
		*S. javaense* Takaoka & Hadi	(4)	0.22 (0.44)
	*S. multistriatum*	*S. bullatum* Takaoka & Choochote	2	–
		*S. chainarongi* Kuvangkadilok & Takaoka	3	0
		*S. chaliowae* Takaoka & Boonkemtong	2	–
		*S. fenestratum* Edwards	6 (1)	2.58 (4.42)^3^
		*S. ubonae* Thaijarern, Wongpakam, Kangrang & Pramual	2	–
	*S. nebulicola*	*S. nebulicola* Edwards	(1)	–
	*S. nobile*	*S. nobile* De Meijere	5 (4)	0.82 (1.99)
		*S. timorense* Takaoka, Hadi & Sigit	4 (4)	0.26 (0.66)
		*S. vanluni* Ya’cob, Takaoka & Sofian-Azirun	5	0.49 (0.88)
	*S. striatum*	*S. argyrocinctum* De Meijere	(4)	2.80 (3.54)
		*S. baliense* Takaoka & Sofian-Azirun	(1)	–
		*S. chiangmaiense* Takaoka & Suzuki	4	0.88 (1.33)
		*S. nakhonense* Takaoka & Suzuki	2	–
		*S. wangkwaiense* Takaoka, Srisuka & Saeung	2	–
	*S. tuberosum*	*S. jianshiense* Takaoka, Otsuka & Adler	3	0.44 (0.44)
		*S. keningauense* Takaoka	(4)	0.22 (0.44)
		*S. tani* Takaoka & Davies	5 (1)	5.32 (7.74)^3^

^3^The intraspecific divergences indicate possible presence of cryptic species

Interspecific genetic distances ranged from 0 to 19.25%, with an average of 13.22%. Low levels of minimum interspecific distance were noted in the following species pairs, suggesting that the individuals of the two species in each pair are closely related or perhaps conspecific: *S.* (*N.*) *aureohirtum* and *S.* (*N.*) *wayani* (0.66%), *S.* (*S.*) *iridescens* and *S.* (*S.*) *javaense* (0.66%), *S.* (*S.*) *chainarongi* and *S.* (*S.*) *ubonae* (0.88%), *S.* (*S.*) *chaliowae* and *S.* (*S.*) *fenestratum* (0.22%), and *S.* (*S.*) *fenestratum* and *S.* (*S.*) *ubonae* (0.66%). Table S1 shows the intraspecific and interspecific genetic distances of each species (see Additional file 2).

### Species delimitation analyses

For ASAP analysis, a few subsequent partitions other than the “best” one with the lowest ASAP score and the threshold distance were considered while choosing the final species partition [37]. The fifth partition with an ASAP score of 11 and threshold distance of 0.034 was chosen among the 10 “best” partitions found by the ASAP analysis using a simple distance substitution model. The distance-based ASAP method and GMYC revealed comparable results, which were 44 and 42, respectively, whereas the single PTP method revealed 51 operational taxonomic units (OTUs). Overall, all three species delimitation analyses showed good agreement, although the single PTP method identified more putative species than did the other two methods. The non-monophyletic groups, such as the *S.* (*N.*) *feuerborni* and *S.* (*S.*) *multistriatum* groups, were considered by the analyses as single taxonomic units, with their members inseparable. Also, more than one taxonomic unit was detected within the single species that had high intraspecific distances (> 3%), except for *S.* (*S.*) *iridescens*.

### Species identification efficacy

The percentages of correct species identifications via the best match and best close match methods exceeded 80% (Table 3). Incorrect identifications were associated with non-monophyletic species as follows: *S.* (*N.*) *aureohirtum, S.* (*N.*) *feuerborni, S.* (*N.*) *fruticosum, S.* (*N.*) *pumatense, S.* (*S.*) *argyrocinctum, S.* (*S.*) *fenestratum, S.* (*S.*) *iridescens* and *S.* (*S.*) *nakhonense.* Lack of conspecifics in database might also cause ambiguous and incorrect identifications of the following species: *S.* (*G.*) *johorense, S.* (*G.*) *laosense, S.* (*G.*) *rangatense, S.* (*G.*) *sumbaense, S.* (*G.*) *sunapii, S.* (*S.*) *baliense* and *S.* (*S.*) *nebulicola.*

**Table 3:** COI identifications of black flies based on best match (BM) and best close match (BCM) methods

Species identification methods	Correct identifications % (*n*)	Ambiguous % (*n*)	Incorrect identifications % (*n*)	Sequences w/o any match closer than 1.9% (*n*)
Best match	89.7 (183)	0.5 (1)	9.8 (20)	–
Best close match	83.8 (171)	0.0 (0)	3.9 (8)	12.3 (25)

## Discussion

The relationships among 55 nominal species of black flies in 14 previously established species groups in Indonesia are presented for the first time to our knowledge through DNA barcodes based on the mitochondrial COI gene. The accuracy of the COI gene to identify black fly species in Indonesia is > 84%. Most of the species are shown to be monophyletic in their respective species groups and subgenera with a few exceptions. Possible causes of non-monophyly include inadequate phylogenetic signal, imperfect taxonomy, interspecific hybridization, incomplete lineage sorting and gene paralogy [44].

In the *S. batoense* group, *S.* (*G.*) *tahanense* forms a single group distinct from other group members. This topology agrees with previous phylogenetic analyses [45, 46]. In fact, *S.* (*G.*) *tahanense* is distinctive not only among species of *S. batoense* species group but also among species of the subgenus *Gomphostilbia* by having the elongate female labrum [47]. The unique characteristic observed in *S.* (*G.*) *tahanense* is believed to contribute to its distinctiveness from other taxa. The grouping of *S.* (*G.*) *rangatense* of the *S. ceylonicum* group with *S.* (*G.*) *sunapii* causes the *S. asakoae* group to be non-monophyletic. Even so, a high genetic distance of 8.85% was recorded between these two species, each of which is recognized as a distinct species. The grouping might be due to inadequate phylogenetic signal of the COI gene in resolving the two species groups, as shown by Low, Takaoka [48].

*Simulium* (*Gomphostilbia*) *sheilae* from Indonesia is probably a distinct lineage from this nominal species in Malaysia and Thailand, based on our results. In the barcode tree, *S.* (*G.*) *sheilae* is divided into three subclades: (i) Indonesia and Malaysia; (ii) Thailand; (iii) Indonesia, which are regarded as different taxonomic units by the delimitation analyses. Furthermore, *S.* (*G.*) *sheilae* from Indonesia displayed high intraspecific distances (minimum = 3.10%) compared to lineages from Malaysia and Thailand. Conversely, a single sample from Indonesia showed a high genetic distance (minimum = 8.63%) compared to other Indonesian sequences, indicating a high level of intraspecific divergence within *S.* (*G.*) *sheilae* in Indonesia. These findings suggest that *S.* (*G.*) *sheilae* in Indonesia may harbour cryptic diversity. *Simulium* (*Gomphostilbia*) *trangense* also has a lower genetic distance from *S.* (*G.*) *sheilae* from Indonesia (minimum = 1.77%) than from Malaysia (minimum = 9.96%) and Thailand (minimum = 6.19%), indicating that *S.* (*G.*) *trangense* is genetically more closely related to *S.* (*G.*) *sheilae* from Indonesia.

*Simulium* (*Nevermannia*) *feuerborni* is a species complex of four chromosomally distinct lineages from Thailand (cytoforms A and B), Malaysia (cytoform C, subsequently named *S.* (*N.*) *pairoti*) and Indonesia (cytoform D), although molecular analysis was not conducted on the Indonesian population in the original studies [23, 24, 26]. Our study supports the distinctiveness of the Indonesian lineage with high divergence values (minimum = 9.29%) reported between Indonesian and Thai lineages. The two lineages are also considered different taxonomic units. Besides, one sequence of Indonesian *S.* (*N.*) *feuerborni* (GenBank accession number: KX525228) has high genetic distance of 5.09% against other Indonesian sequences. Moreover, ASAP and PTP analyses also detected two taxonomic units in the Indonesian *S.* (*N.*) *feuerborni*. These genetic results suggest possible cryptic diversity, though further research is needed to clarify these observations.

Similar to the studies by Thaijarern, Sopaladawan [49] and Pramual, Jomkumsing [20], *S.* (*N.*) *aureohirtum* in our study was divided into two lineages, considered different taxa, that are genetically different, with a maximum distance of 7.96%. However, no evidence was found of sibling species in *S.* (*N.*) *aureohirtum* in Thailand [50]. Further analyses are required to determine whether the two lineages are different species [20]. More specimens of *S.* (*N.*) *aureohirtum* from Indonesia should be included in analyses to determine intraspecific variation and genetic relationships with other taxa. In addition, comparisons with *S.* (*N.*) *aureohirtum* from the type locality (Assam, India) are essential in sorting out the taxonomy of this nominal species.

The sequences of *S.* (*N.*) *wayani* were nested within one of the *S.* (*N.*) *aureohirtum* subgroups with low genetic distances (minimum = 0.66%), although *S.* (*N.*) *aureohirtum* is readily distinguished from *S.* (*N.*) *wayani* by the number of pupal gill filaments, suggesting that *S.* (*N.*) *wayani* is closely related to the *S.* (*N.*) *aureohirtum* subgroup. Chromosomal analyses indicate, however, that *S.* (*N.*) *wayani* is closely related to the *S.* (*N.*) *ornatipes* complex of mainland Australia [1], indicating that further barcode studies should include the *S.* (*N.*) *ornatipes* complex. Takaoka [51] inferred that species of the *S. ruficorne* group dispersed eastward from Sumatra in Indonesia to the Australasian Region while reducing the pupal gill filaments from eight (*S.* (*N.*) *glattharri* Takaoka & Davies) to four (*S.* (*N.*) *ornatipes*) through six (*S.* (*N.*) *aureohirtum*). *Simulium* (*N.*) *wayani* has four pupal gill filaments. Our results support the hypothesis that *S.* (*N.*) *wayani* might have evolved from an ancestral six-filamented population of *S.* (*N.*) *aureohirtum*, proposed by Takaoka, Sofian-Azirun [52], perhaps along with members of the *S.* (*N.*) *ornatipes* complex [1].

As expected from Pramual and Nanork [53], *S.* (*S.*) *fenestratum* was paraphyletic with respect to other members of the *S. multistriatum* group. The specimen from Indonesia forms a clade separate from the Thailand sequences retrieved from GenBank, although Indonesian *S.* (*S.*) *fenestratum* is genetically closer to two Thailand sequences (GenBank accession numbers: MG734051 and MG734055). The intraspecific variation of *S.* (*S.*) *fenestratum* from Indonesia could not be examined, as only one specimen was available. *Simulium* (*Simulium*) *ubonae* has low interspecific distances compared with other taxa in our study. The genetic distances of *S.* (*S.*) *ubonae* compared with those of *S.* (*S.*) *chainarongi* (0.88%) and one sequence of *S.* (*S.*) *fenestratum* (0.66%) are especially low, indicating *S.* (*S.*) *ubonae* is genetically closer to these two species. This result does not agree with a previous study showing high interspecific distances (minimum = 4.9%) of *S. (S.)* *ubonae* [54]. The non-monophyly of *S.* (*S.*) *chiangmaiense, S.* (*S.*) *nakhonense* and *S.* (*S.*) *wangkwaiense* in the *S. striatum* group in our study was expected; a previous study by Pangjanda and Pramual [55] showed that the COI gene was unable to separate these three taxa.

In the *S. tuberosum* group, *S.* (*S.*) *tani* is a large species complex [56–58]; thus, the high intraspecific divergence in our study was expected. Although the single barcode of *S.* (*S.*) *tani* showed high intraspecific distances (minimum = 3.10%) compared to other Thailand sequences, delimitation methods do not classify *S.* (*S.*) *tani* from Indonesia as a separate taxonomic unit. However, due to the availability of only one sample, genetic results provide limited information on the intraspecific variation of *S.* (*S.*) *tani* from Indonesia.

A rough indicator of separate species in the Simuliidae has been suggested as 3% divergence [59]. Accordingly, *S.* (*G.*) *gyorkosae, S.* (*G.*) *cheongi, S.* (*G.*) *floresense, S.* (*S.*) *eximium, S.* (*S.*) *iridescens and S.* (*S.*) *argyrocinctum* are possible species complexes. All COI sequences of these nominal species, except *S.* (*G.*) *cheongi*, are reported here for the first time. Takaoka and Davies [15] first suspected that *S.* (*G.*) *iridescens* is a species complex because males from West Java differ from those at the type locality in East Java. Morphological differences have also been found between males of *S.* (*G.*) *gyorkosae* from Bali and Lombok [60]. The cytotaxonomy of *S.* (*S.*) *eximium* suggested that it includes two cryptic species [61]. For *S.* (*G.*) *floresense* and *S.* (*S.*) *argyrocinctum*, no morphological or cytogenetic studies indicate possible cryptic diversity. Intraspecific distances of these species, which exceed 3%, hint at possible cryptic diversity, but more study is required. On the other hand, the COI gene strongly suggests that *S.* (*G.*) *cheongi* from Indonesia and Malaysia represents two genetically distinct species, as evidenced by the high genetic divergence between the two lineages and their placements in the tree. The two clades are also recognised as separate taxa. The Malaysian lineage is more closely related to *S.* (*G.*) *atratum* based on their genetic distance and the sister relationship between the two species.

In addition to the species pairs with low levels of interspecific distances described earlier, the two species in the following species pairs group together in the tree and possess low minimum genetic distances between them: *S.* (*G.*) *sumbaense* and *S.* (*G.*) *chumpornense* (2.21%), *S.* (*S.*) *nobile* and *S.* (*S.*) *timorense* (1.11%), and *S.* (*S.*) *baliense* and *S.* (*S.*) *argyrocinctum* (2.21%)*.* The low interspecific distances between *S.* (*S.*) *nobile* and *S.* (*S.*) *timorense* are comparable to those in previous studies [27, 29]. *Simulium* (*Gomphostilbia*) *sumbaense* is assigned to the *S. chumpornense* subgroup and has a similar arrangement of pupal gill filaments to *S.* (*G.*) *chumpornense* [52]. In contrast, *S.* (*S.*) *baliense* and *S.* (*S.*) *argyrocinctum* are structurally alike in their pupal gill arrangements [60]. Although these three species pairs are structurally alike, the species are nonetheless separable by other characters. Their low genetic distances suggest that the members of each pair are closely related.

## Conclusions

COI-based DNA barcoding is a valuable means of identification of black flies in Indonesia, except for a limited number of taxa, especially nominal species known to be complexes. The separation of these problematic taxa requires other options, such as fast-evolving genes and cytogenetics. Several nominal species were unavailable for in-depth inspection because of limited sampling. For instance, only one sequence was included for the following species, limiting the study of their intraspecific variation: *S.* (*G.*) *sunapii, S.* (*G.*) *rangatense, S.* (*G.*) *sumbaense, S.* (*N.*) *aureohirtum, S.* (*S.*) *fenestratum, S.* (*S.*) *nebulicola, S.* (*S.*) *baliense* and *S.* (*S.*) *tani*. Therefore, more samples should be collected from Indonesia for in-depth studies. Furthermore, no morphological variation was observed in the species that showed high intraspecific divergences; further detailed morphological examinations are thus required to confirm the presence of cryptic diversity. Nevertheless, this research provides a basis for future comprehensive studies on black flies in Indonesia. The deposition of COI sequences into publicly accessible databases also enables the establishment of a novel sequence library for Indonesian black flies. Additionally, the nucleotide database is expected to serve as a reference for species identification and comparative studies of other species of Indonesian black flies that were not included in this study. Overall, our findings establish the groundwork for further utilization of COI barcoding as a rapid and precise method for exploring the diversity of Indonesian black flies.

## Supplementary Information

**Additional file 1: Figure. S1**. BI tree showing species of black flies from Indonesia in the subgenus *Simulium* Latreille, *Nevermannia *Enderlein and *Gomphostilbia *Enderlein, which was constructed from COI sequences. Posterior probability values of > 0.50 are shown on the branches. Branches with posterior probability values > 0.70 are considered well supported. New sequences generated in the study are in bold. Grey bars indicate the respective operational taxonomic units recognised by the three species delimitation analyses (i.e. ASAP, GMYC and PTP, in order). For GMYC analysis, the three bars labelled with double asterisks (**) represent one taxonomic unit, while the two bars labelled with hashtag (#) symbols represent another taxonomic unit. ASAP: Assemble Species by Automatic Partitioning; GMYC: Generalized Mixed Yule Coalescent; PTP: Poisson Tree Processes

**Additional file 2: Table S1**. Intra- and interspecific genetic distances of Indonesian black flies (*n* = 55) included in the study, calculated based on uncorrected p-distance method.

## Data Availability

Representative COI sequences were deposited into the NCBI GenBank database under accession numbers OQ117897–OQ117982 and the Global Biodiversity Information Facility (GBIF) database, which is available at https://www.gbif.org/dataset/c80987f7-f87a-4ae3-a2cc-ccd59bc951e8.

## Abbreviations

ASAP: Assemble Species by Automatic Partitioning
BCM: Best close match
BM: Best match
COI: Cytochrome *c* oxidase subunit I
GMYC: Generalized Mixed Yule Coalescent
mPTP: Multi-rate Poisson Tree Processes
PCR: Polymerase chain reaction
PTP: Poisson Tree Processes

## References

[1] Adler PH, Takaoka H, Sofian-Azirun M, Chen CD, Suana IW (2019). Evolutionary and biogeographic history of the black fly *Simulium wayani* (Diptera: Simuliidae) on the island of Timor. Acta Trop.

[2] Adler PH, McCreadie JW, Adler PH, McCreadie JW (2019). Black flies (Simuliidae). Medical and veterinary entomology.

[3] Thaijarern J, Tangkawanit U, Wongpakam K, Pramual P (2019). Molecular detection of *Trypanosoma* (Kinetoplastida: Trypanosomatidae) in black flies (Diptera: Simuliidae) from Thailand. Acta Trop.

[4] Malmqvist B, Adler PH, Kuusela K, Merritt RW, Wotton RS (2004). Black flies in the boreal biome, key organisms in both terrestrial and aquatic environments: a review. Ecoscience.

[5] Pramual P, Petney TN, Saijuntha W, Mehlhorn H (2021). Black fly diversity and impacts on human welfare in Southeast Asia. Biodiversity of Southeast Asian parasites and vectors causing human disease.

[6] Adler PH. World blackflies (Diptera: Simuliidae): a comprehensive revision of the taxonomic and geographical inventory [2022] 2022.

[7] Hadi UK, Takaoka H (2018). The biodiversity of black flies (Diptera: Simuliidae) in Indonesia. Acta Trop.

[8] Friederichs K (1925). Beobachtungen an simuliiden in ost-java. Archiv fur Schiffs-und Tropenhygiene.

[9] Takaoka H, Noda S (1979). Autogeny of the black fly *Simulium (Eusimulium) aureohirtum* (Diptera: Simuliidae). J Med Entomol.

[10] Takaoka H (1989). Further observations on the autogeny of *Simulium aureohirtum* Brunetti (Diptera: Simuliidae) in the Ryukyu Islands. Med Entomol Zool.

[11] Adler PH, Currie DC, Wood DM (2004). The black flies (Simuliidae) of North America.

[12] Crosskey R (1967). The classification of *Simulium* latreille (Diptera: Simuliidae) from Australia, New Guinea and the Western Pacific. J Nat Hist.

[13] Shelley AJ (2010). Blackflies (Diptera: Simuliidae) of Brazil.

[14] Takaoka H (2003). The black flies (Diptera: Simuliidae) of sulawesi maluku and irian jaya.

[15] Takaoka H, Davies DM (1996). The black flies (Diptera: Simuliidae) of java, Indonesia. Bishop Mus Bull Entomol.

[16] Takaoka H, Davies DM (1995). The black flies (Diptera: Simuliidae) of west Malaysia.

[17] Takaoka H, Sofian-Azirun M, Yacob Z, Chen CD, Lau KW, Low VL (2017). The black flies (Diptera: Simuliidae) of Vietnam. Zootaxa.

[18] Conflitti I, Pruess K, Cywinska A, Powers TO, Currie D (2013). DNA barcoding distinguishes pest species of the black fly genus *Cnephia* (Diptera: Simuliidae). J Med Entomol.

[19] Rivera J, Currie DC (2009). Identification of nearctic black flies using DNA barcodes (Diptera: Simuliidae). Mol Ecol Resour.

[20] Pramual P, Jomkumsing P, Wongpakam K, Wongwian P (2021). DNA barcoding of tropical black flies (Diptera: Simuliidae) in Thailand: one decade of progress. Acta Trop.

[21] Pramual P, Adler PH (2014). DNA barcoding of tropical black flies (Diptera: Simuliidae) of Thailand. Mol Ecol Resour.

[22] Pramual P, Kuvangkadilok C (2012). Integrated cytogenetic, ecological, and DNA barcode study reveals cryptic diversity in *Simulium (Gomphostilbia) angulistylum* (Diptera: Simuliidae). Genome.

[23] Pramual P, Wongpakam K (2013). Population genetics of the high elevation black fly *Simulium (Nevermannia) feuerborni* edwards in Thailand. Entomol Sci.

[24] Pramual P, Thaijarern J, Sofian-Azirun M, Yacob Z, Hadi UK, Takaoka H (2015). Cytogenetic and molecular evidence of additional cryptic diversity in high elevation black fly *Simulium feuerborni* (Diptera: Simuliidae) populations in Southeast Asia. J Med Entomol.

[25] Thaijarern J, Adler PH, Pramual P (2018). Limited differentiation among black flies in the *Simulium multistriatum* species group (Diptera: Simuliidae) in Thailand: cryptic species, homosequential species and homosequential cryptic species. Zool J Linn Soc.

[26] Ya’cob Z, Takaoka H, Low VL, Sofian-Azirun M (2017). Uncovering the mask of the *Simulium feuerborni* complex (Diptera: Simuliidae): description of a new pseudocryptic species *Simulium pairoti* from Malaysia. Acta Trop.

[27] Ya’cob Z, Takaoka H, Low VL, Sofian-Azirun M (2017). First description of a new cryptic species, *Simulium vanluni* from Peninsular Malaysia: an integrated morpho-taxonomical and genetic approach for naming cryptic species in the family Simuliidae. Acta Trop.

[28] Adler PH, Fukuda M, Takaoka H, Reeves WK, Kim S-K, Otsuka Y (2019). Revision of *Simulium rufibasis* (Diptera: Simuliidae) in Japan and Korea: chromosomes, DNA, and morphology. J Med Entomol.

[29] Low VL, Takaoka H, Pramual P, Adler PH, Ya’cob Z, Chen CD (2016). Three taxa in one: cryptic diversity in the black fly *Simulium nobile* (Diptera: Simuliidae) in Southeast Asia. J Med Entomol.

[30] Low VL, Adler PH, Sofian-Azirun M, Srisuka W, Saeung A, Huang Y-T (2015). Tests of conspecificity for allopatric vectors: *Simulium nodosum* and *Simulium shirakii* (Diptera: Simuliidae) in Asia. Parasit Vectors.

[31] Folmer O, Black M, Hoeh W, Lutz R, Vrijenhoek R (1994). DNA primers for amplification of mitochondrial cytochrome *c* oxidase subunit I from diverse Metazoan invertebrates. Mol Mar Biol Biotech.

[32] Okonechnikov K, Golosova O, Fursov M (2012). Unipro UGENE: a unified bioinformatics toolkit. Bioinformatics.

[33] Hall TA (1999). BioEdit: a user-friendly biological sequence alignment editor and analysis program for windows 95/98/NT.

[34] Tanabe AS (2011). Kakusan4 and Aminosan: two programs for comparing nonpartitioned, proportional and separate models for combined molecular phylogenetic analyses of multilocus sequence data. Mol Ecol Resour.

[35] Kozlov AM, Darriba D, Flouri T, Morel B, Stamatakis A (2019). RAxML-NG: a fast, scalable and user-friendly tool for maximum likelihood phylogenetic inference. Bioinformatics.

[36] Ronquist F, Teslenko M, van der Mark P, Ayres DL, Darling A, Höhna S (2012). MrBayes 32: efficient bayesian phylogenetic inference and model choice across a large model space. Syst Biol.

[37] Puillandre N, Brouillet S, Achaz G (2021). ASAP: assemble species by automatic partitioning. Mol Ecol Resour.

[38] Pons J, Barraclough TG, Gomez-Zurita J, Cardoso A, Duran DP, Hazell S (2006). Sequence-based species delimitation for the DNA taxonomy of undescribed insects. Syst Biol.

[39] Zhang J, Kapli P, Pavlidis P, Stamatakis A (2013). A general species delimitation method with applications to phylogenetic placements. Bioinformatics.

[40] Ezard T, Fujisawa T, Barraclough T. splits: SPecies’ LImits by Threshold Statistics. R package version 1.0-14/r31. 2009.

[41] Tamura K, Stecher G, Kumar S (2021). MEGA11: molecular evolutionary genetics analysis version 11. Mol Biol Evol.

[42] Meier R, Shiyang K, Vaidya G, Ng PKL (2006). DNA barcoding and taxonomy in Diptera: a tale of high intraspecific variability and low identification success. Syst Biol.

[43] Sonet G, Jordaens K, Nagy Z, Breman F, Meyer M, Backeljau T (2013). *Adhoc*: an R package to calculate *ad hoc* distance thresholds for DNA barcoding identification. ZooKeys.

[44] Funk DJ, Omland KE (2003). Species-level paraphyly and polyphyly: frequency, causes, and consequences, with insights from animal mitochondrial DNA. Annu Rev Ecol Evol Syst.

[45] Takaoka H, Srisuka W, Van Lun L, Saeung A (2018). Five new species of the *Simulium decuplum* subgroup of the *Simulium (Gomphostilbia) batoense* species-group (Diptera: Simuliidae) from Thailand and their phylogenetic relationships. Acta Trop.

[46] Takaoka H, Fukuda M, Otsuka Y, Low VL, Ya’cob Z (2022). Redescription of *Simulium* (*Gomphostilbia*) *omutaense* Ogata & Sasa (Diptera: Simuliidae) from Japan and its phylogenetic relationship with other members of the *S. batoense* species-group. Acta Trop.

[47] Takaoka H (2012). Morphotaxonomic revision of *Simulium* (*Gomphostilbia*) (Diptera: Simuliidae) in the Oriental Region. Zootaxa.

[48] Low VL, Takaoka H, Adler PH, Ya'cob Z, Norma-Rashid Y, Chen CD (2015). A multi-locus approach resolves the phylogenetic relationships of the *Simulium asakoae* and *Simulium ceylonicum* species groups in Malaysia: evidence for distinct evolutionary lineages. Med Vet Entomol.

[49] Thaijarern J, Sopaladawan PN, Wongpakam K, Pramual P (2014). Phylogeography of the black fly *Simulium aureohirtum* (Diptera: Simuliidae) in Thailand. Genome.

[50] Pramual P, Wongpakam K, Kuvangkadilok C (2008). Cytogenetics of the black fly *Simulium aureohirtum* Brunetti from Thailand. Cytologia.

[51] Takaoka H (2017). Speciation, faunal affinities and geographical dispersal of black flies (Diptera: Simuliidae) in the Oriental Region. Acta Trop.

[52] Takaoka H, Sofian-Azirun M, Chen CD, Lau KW, Halim M, Low VL (2018). Three new species of black flies (Diptera: Simuliidae) from the lesser sunda archipelago, Indonesia. Trop Biomed.

[53] Pramual P, Nanork P (2012). Phylogenetic analysis based on multiple gene sequences revealing cryptic biodiversity in *Simulium multistriatum* group (Diptera: Simuliidae) in Thailand. Entomol Sci.

[54] Thaijarern J, Wongpakam K, Kangrang A, Pramual P (2019). A new species of black fly (Diptera: Simuliidae) in the *Simulium (Simulium) multistriatum* species-group from Thailand. Zootaxa.

[55] Pangjanda S, Pramual P (2017). Tests of conspecificity for closely related black fly (Diptera: Simuliidae) species of the * Simulium striatum * group in Thailand. Zootaxa.

[56] Adler PH, Huang Y-T, Reeves WK, Kim SK, Otsuka Y, Takaoka H (2013). Macrogenomic evidence for the origin of the black fly *Simulium suzukii* (Diptera: Simuliidae) on Okinawa Island, Japan. PLoS ONE.

[57] Pramual P, Kuvangkadilok C, Baimai V, Walton C (2005). Phylogeography of the black fly *Simulium tani* (Diptera: Simuliidae) from Thailand as inferred from mtDNA sequences. Mol Ecol.

[58] Tangkawanit U, Kuvangkadilok C, Baimai V, Adler PH (2009). Cytosystematics of the *Simulium tuberosum* group (Diptera: Simuliidae) in Thailand. Zool J Linn Soc.

[59] Pramual P, Simwisat K, Martin J (2016). Identification and reassessment of the specific status of some tropical freshwater midges (Diptera: Chironomidae) using DNA barcode data. Zootaxa.

[60] Takaoka H, Sofian-Azirun M, Ya’cob Z, Chen CD, Lau KW, Low VL (2017). The black flies (Diptera: Simuliidae) of the lesser sunda archipelago, Indonesia. Acta Trop.

[61] Hadi UK, Takaoka H, Kondo K, Hirai H (1996). Larval salivary gland chromosomes of *Simulium (Simulium) eximium* (Diptera : Simuliidae) from Java and Sumatra, Indonesia, with implication of sibling speciation. Med Entomol Zool.

