# Supplementary figures and images for: DNA barcoding of black flies (Diptera: Simuliidae) in Indonesia
Source: Parasit Vectors. 2023 Jul 22;16:248. doi: 10.1186/s13071-023-05875-1 (PMC10362752; doi:10.1186/s13071-023-05875-1)

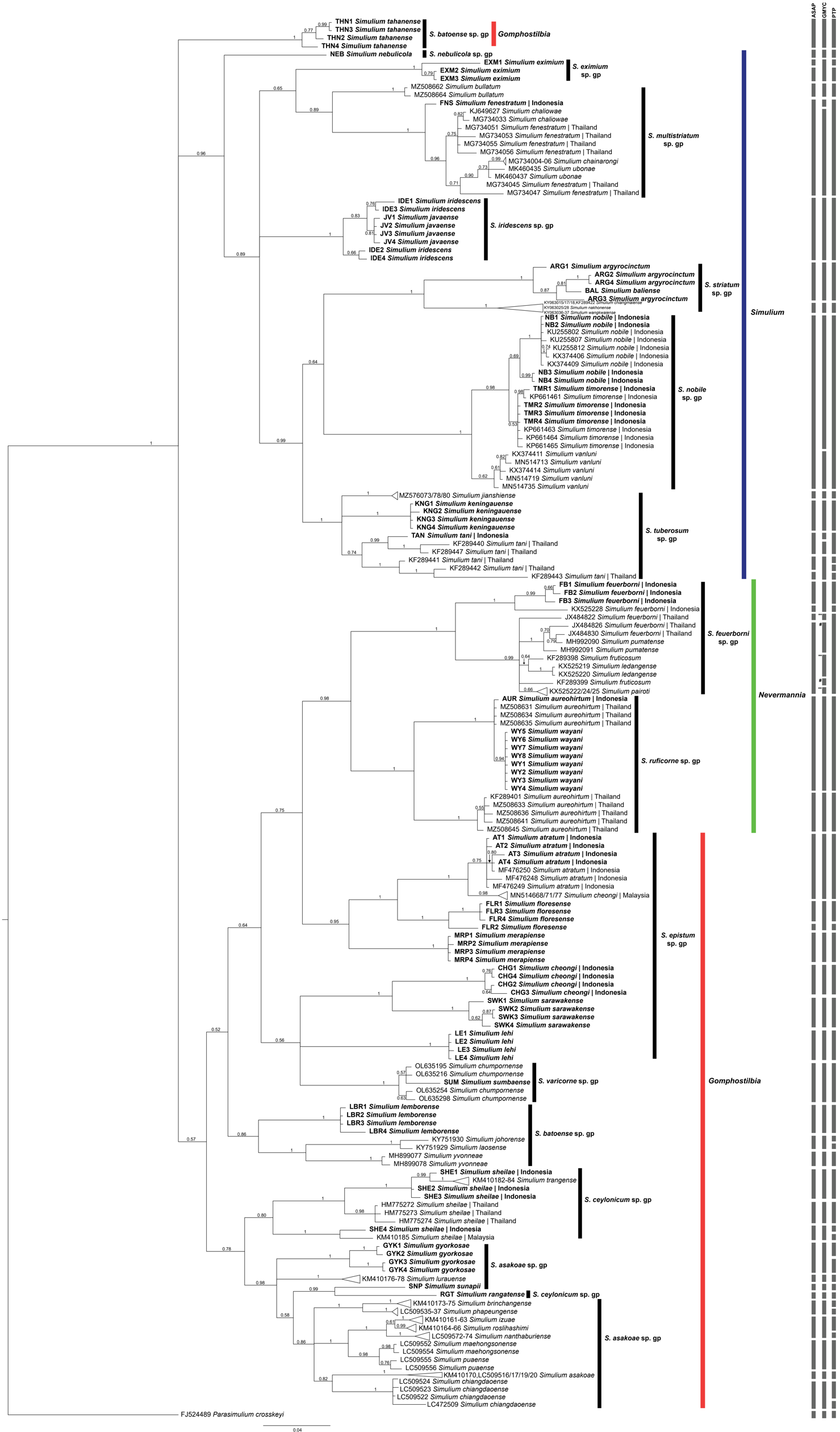

Supplement: Supplementary file 1 — Additional file 1: Figure. S1. BI tree showing species of black flies from Indonesia in the subgenus Simulium Latreille, Nevermannia Enderlein and Gomphostilbia Enderlein, which was constructed from COI sequences. Posterior probability values of > 0.50 are shown on the branches. Branches with posterior probability values > 0.70 are considered well supported. New sequences generated in the study are in bold. Grey bars indicate the respective operational taxonomic units recognised by the three species delimitation analyses (i.e. ASAP, GMYC and PTP, in order). For GMYC analysis, the three bars labelled with double asterisks (**) represent one taxonomic unit, while the two bars labelled with hashtag (#) symbols represent another taxonomic unit. ASAP: Assemble Species by Automatic Partitioning; GMYC: Generalized Mixed Yule Coalescent; PTP: Poisson Tree Processes [file 13071_2023_5875_MOESM1_ESM.pdf]
